# High-Throughput NanoBiT-Based Screening for Inhibitors of HIV-1 Vpu and Host BST-2 Protein Interaction

**DOI:** 10.3390/ijms22179308

**Published:** 2021-08-27

**Authors:** Boye Li, Xiaoxiao Dong, Wenmei Zhang, Tian Chen, Boyang Yu, Wenyue Zhao, Yishu Yang, Xiaoli Wang, Qin Hu, Xiayan Wang

**Affiliations:** 1 The Faculty of Environment and Life, Beijing University of Technology, Beijing 100124, China; liboye@emails.bjut.edu.cn (B.L.); dxx0119@emails.bjut.edu.cn (X.D.); zwm1436271046@emails.bjut.edu.cn (W.Z.); chentian@emails.bjut.edu.cn (T.C.); yuby@emails.bjut.edu.cn (B.Y.); clairezhaooo@163.com (W.Z.); yishu-y@bjut.edu.cn (Y.Y.); wangxiaoli@bjut.edu.cn (X.W.); 2 Center of Excellence for Environmental Safety and Biological Effects, Beijing Key Laboratory for Green Catalysis and Separation, Department of Chemistry and Biology, Beijing University of Technology, Beijing 100124, China; 3 Beijing International Science and Technology Cooperation Base of Antivirus Drug, Beijing University of Technology, Beijing 100124, China

**Keywords:** HIV-1, Vpu, BST-2, NanoLuc Binary Technology, high-throughput screening assay

## Abstract

Bone marrow stromal cell antigen 2 (BST-2), also known as CD317 or tetherin, has been identified as a host restriction factor that suppresses the release of enveloped viruses from host cells by physically tethering viral particles to the cell surface; however, this host defense can be subverted by multiple viruses. For example, human immunodeficiency virus (HIV)-1 encodes a specific accessory protein, viral protein U (Vpu), to counteract BST-2 by binding to it and directing its lysosomal degradation. Thus, blocking the interaction between Vpu and BST-2 will provide a promising strategy for anti-HIV therapy. Here, we report a NanoLuc Binary Technology (NanoBiT)-based high-throughput screening assay to detect inhibitors that disrupt the Vpu-BST-2 interaction. Out of more than 1000 compounds screened, four inhibitors were identified with strong activity at nontoxic concentrations. In subsequent cell-based BST-2 degradation assays, inhibitor Y-39983 HCl restored the cell-surface and total cellular level of BST-2 in the presence of Vpu. Furthermore, the Vpu-mediated enhancement of pesudotyped viral particle production was inhibited by Y-39983 HCl. Our findings indicate that our newly developed assay can be used for the discovery of potential antiviral molecules with novel mechanisms of action.

## 1. Introduction

Acquired immunodeficiency syndrome (AIDS) is a malignant infectious disease caused by human immunodeficiency virus (HIV), which causes severe destruction of immune function and eventually leads to opportunistic infections and tumors. Current antiretroviral drugs are often classified into six main classes: chemokine receptor antagonists, fusion inhibitors, nucleosides/non-nucleoside reverse transcriptase inhibitors, protease inhibitors, and integrase inhibitors. Although the advent and continuous optimization of combination antiretroviral therapy (cART) has significantly reduced the mortality and morbidity due to HIV infection [1,2], many challenges remain to be overcome, including incomplete adherence to ART, adverse effects, and drug resistance [3]. Therefore, there is still a major need for new antiviral interventions that target novel mechanisms of action.

The HIV-1 viral protein U (Vpu) protein is a type I transmembrane protein expressed in the late stage of the viral life cycle [4,5] and a multifunctional accessory protein that facilitates viral egress. Vpu is found to counteract bone marrow stromal cell antigen 2 (BST-2) by downregulating its expression on the cell surface [6,7] and it also targets newly synthesized clusters of differentiation 4 (CD4) in the endoplasmic reticulum to prevent its trafficking to the plasma membrane and the unwanted formation of premature viral gp160/CD4 complex [8,9]. In addition, Vpu forms cation-selective ion channels or pores in lipid bilayers and possibly augments virion release [10]. In recent years, several new functions have been assigned to Vpu, including the downregulation of CD1d, major histocompatibility complex (MHC) molecules, NK, T- and B-cell antigen (NTB-A), and intercellular adhesion molecule 1 (ICAM-1) on the cell surface [11,12,13,14], deregulation of the nuclear factor kappa B (NF-κB) pathway, and modulation of DNA repair machinery [15]. Although an increasing number of novel targets have been identified, Vpu-BST-2 interaction remains the most active area in Vpu-related research.

BST-2 protein (also known as tetherin, CD317, or HM1.24) is a type II transmembrane protein with an N-terminal cytoplasmic domain and a C-terminal glycosyl-phosphatidylinositol (GPI) anchor. Due to its unique topology, BST-2 physically crosslinks the virions to the surface of infected cells, thereby suppressing the release of newly produced virus particles [16]. It exerts broad-spectrum activities against a variety of enveloped viruses and can be antagonized by different viral proteins, including HIV-1 Vpu [17,18]. The Vpu protein binds to BST-2 via interaction with the transmembrane (TM) region and induces BST-2 ubiquitination through an endosome-lysosome degradation pathway [19]. In addition to enhancing virus release, Vpu counteracts the role of BST-2 in interferon production and antibody-dependent cellular cytotoxicity [20,21]. Therefore, the Vpu-BST-2 interaction is a potential therapeutic target for the development of novel anti-HIV-1 treatments. In recent years, several agents have been identified [22,23,24], but no treatment has yet been approved.

In the present study, we used NanoLuc Binary Technology (NanoBiT) [25] to configure a cell-based Vpu-BST-2 binding assay for high-throughput small-molecule screening. We identified four inhibitors out of 1177 compounds from the immunology/inflammation compound library. They were further tested for activity on Vpu-induced BST-2 degradation and Vpu-mediated pseudotyped viral particle production.

## 2. Results

### 2.1. Development of NanoBiT Assay for Detecting Vpu-BST-2 Interactions

**Figure 1:**
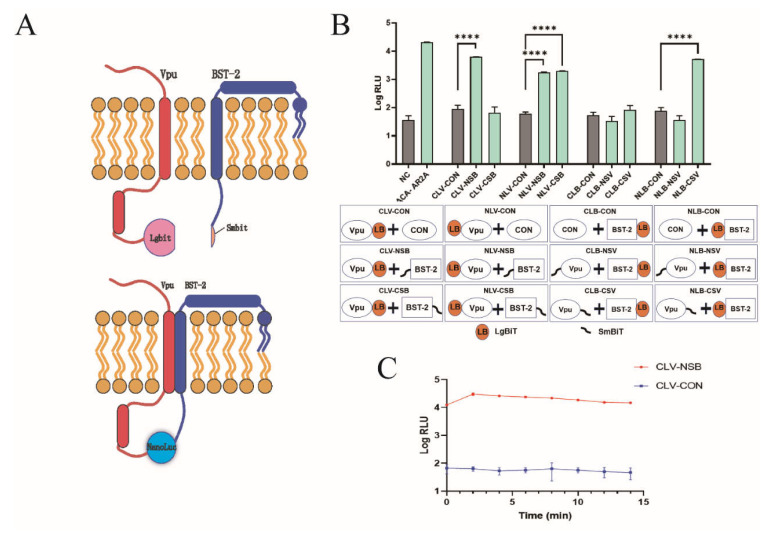
Development of the NanoBiT assay for detecting Vpu-BST-2 interaction. (**A**) Illustrations of the NanoBiT assay to monitor Vpu-BST-2 interaction. (**B**) Optimization of Vpu and BST-2 plasmid combinations. HEK293T cells were co-transfected with eight plasmid combinations as well as their respective controls. Two days later, the fluorescence values were measured to find the combination with the highest luciferase activity. ****, *p* < 0.0001 vs. control group. (NLB: pBiT1.1-N-bst-2; NSB: pBiT2.1-N-bst-2; CLB: pBiT1.1-C-bst-2; CSB: pBiT2.1-C-bst-2; NLV: pBiT1.1-N-vpu; NSV: pBiT2.1-N-vpu; CLV: pBiT1.1-C-vpu; CSV: pBiT2.1-C-vpu; NC: negative control; CON: SmBiT vector control; ACA-AR2A: PRKACA: PRKAR2A pair was used as a constitutive positive control) (**C**) HEK293T cells were co-transfected with plasmids CLV and NSB. Two days later, after adding the substrate, the detection was carried out every 2 min to determine the optimal timepoint of NanoBiT assay. Values presented are the mean ± standard deviation (SD) of three replicates. All experiments were performed five times.

NanoBiT is a luciferase-based complementation reporter system that is widely used for the quantitative analysis of protein–protein interactions. The NanoBiT system is composed of a large (LgBiT, 18 kDa) and small BiT subunit (SmBiT, 1.3 kDa). In our study, Vpu and BST-2 proteins were fused to LgBiT and SmBiT, respectively, and co-expressed in HEK293T cells. When Vpu binds to BST-2, LgBiT and SmBiT subunits are brought together, resulting in the formation of NanoLuc luciferase (NLuc), which can be quantitatively measured and is applicable to high-throughput screening (Figure 1A). To optimize the orientation of LgBiT and SmBiT on Vpu and BST-2, respectively, we constructed eight pairs of Vpu and BST-2 containing vectors with either LgBiT or SmBiT fused to the N- or C-terminus. Following co-transfection into HEK293T cells, the luminescence signal was detected in all Vpu/BST-2 pairs and the highest luciferase activity was observed in CLV-NSB (Vpu with C-terminal LgBiT and BST-2 with N-terminal SmBiT), which generated a 95-fold increase over the corresponding control (Vpu with C-terminal LgBiT and HaloTag with SmBiT) as shown in Figure 1B. This combination was selected for the follow-up experiments. In addition, we detected the half-life of the luminescence signal and found that the signal of CLV-NSB increased in the first 2 min and decreased slightly afterward; it remained 100-fold higher than that of the control after 15 min (Figure 1C). This indicates that the luminescence signal was highly stable during the first 15 min.

### 2.2. Identification of Inhibitors Targeting Vpu-BST-2 Interactions

**Figure 2:**
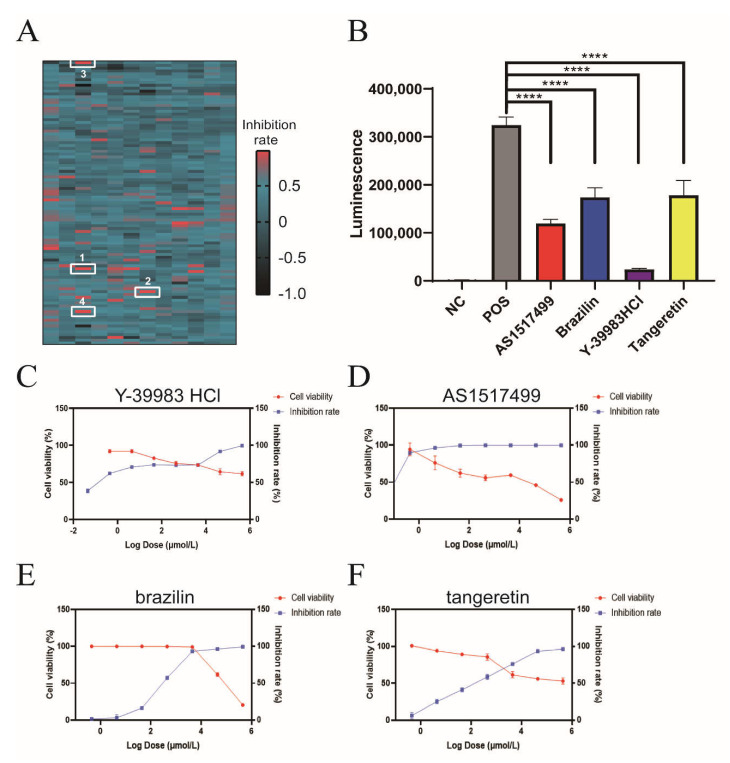
High-throughput compound screening by NanoBiT assay. (**A**) Composite heat map of 1177 compounds screened by NanoBiT assay. HEK293T Cells were co-transfected with plasmids CLV (Vpu with C-terminal LgBiT) and NSB (BST-2 with N-terminal SmBiT). Compounds with a final concentration of 10 μmol/L were added 4 h later. The heat map is drawn from the calculated inhibition rate. AS1517499 (1), Y-39983 HCl (2), brazilin (3) and tangeretin (4) were labeled with white rectangles. (**B**) HEK293T cells were co-transfected with plasmids CLV and NSB and then treated with Y-39983 HCl, brazilin, tangeretin, or AS1517499 (10 μmol/L), respectively. Luciferase activities were measured using a multilabel reader. ****, *p* < 0.0001 vs. postive control group. Dose-dependent curves of the inhibition rate and cell viability of HEK293T cells treated with (**C**) Y-39983 HCl, (**D**) AS1517499, (**E**) brazilin, or (**F**) tangeretin. Values presented are the mean ± SD of three replicates.

For the first round of screening, a total of 1177 compounds were initially screened for inhibitory activity on Vpu-BST-2 interactions, and 21 hits were identified with an inhibition rate greater than 85% (Figure 2A). In the confirmatory repeat screening, only four compounds (Y-39983 HCl, brazilin, tangeretin, and AS1517499) significantly inhibited the luminescence signal at 10 μmol/L (Figure 2B), and they were further tested in titrations to evaluate the cytotoxicity and activity of Vpu-BST-2 binding in parallel. The therapeutic indexes (CC_50_/EC_50_) for Y-39983 HCl, brazilin, tangeretin, and AS1517499 were >91, 4.5, >14.8, and 47, respectively (Figure 2C–F and Table S1), indicating an obvious binding inhibition efficacy and low cytotoxicity of compounds.

### 2.3. Protective Activity of Compounds against Vpu-Induced BST 2 Degradation

**Figure 3:**
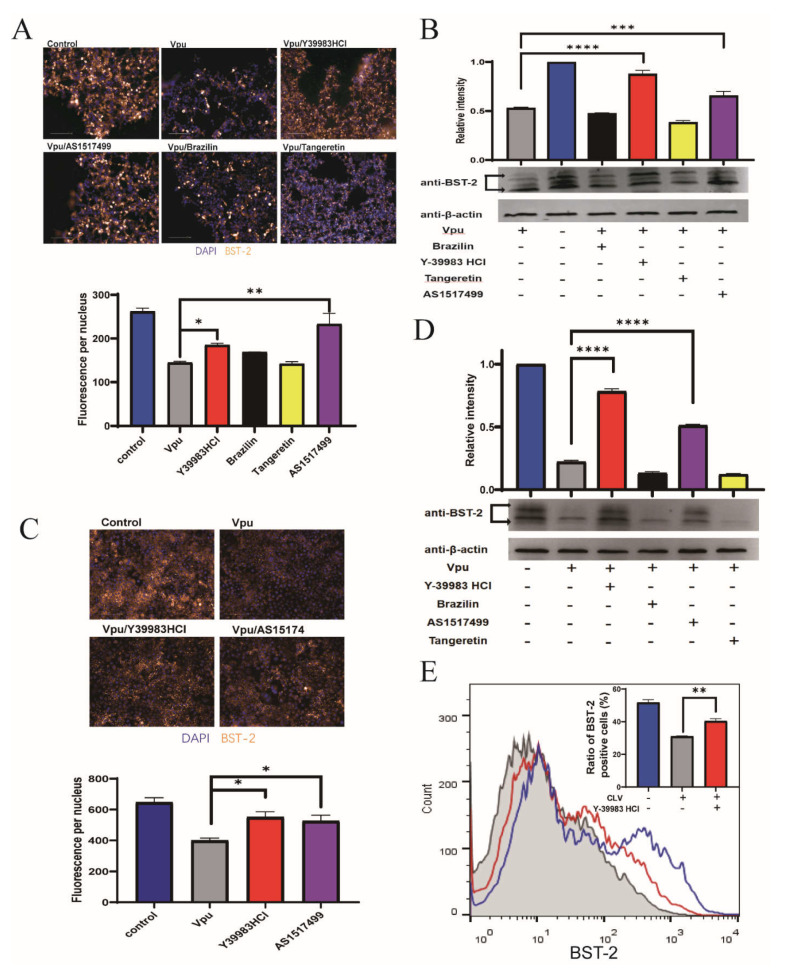
Compounds inhibited Vpu-mediated downregulation of BST-2. HEK293T cells were co-transfected with plasmids CLV (Vpu with C-terminal LgBiT) and NSB (BST-2 with N-terminal SmBiT), and treated with Y-39983 HCl, brazilin, tangeretin, or AS1517499 (10 μmol/L) for 36 h. Total cellular expression of BST-2 was measured using a high-content imaging system (**A**) and Western blotting (**B**). (**C**) HeLa cells were transfected with CLV vector and treated with Y-39983 HCl, brazilin, tangeretin, or AS1517499 (10 μmol/L) for 36 h. Total cellular expression of BST-2 was measured by high-content imaging analysis and Western blotting (**D**). (**E**) HEK293T cells were co-transfected with plasmids CLV and NSB, and treated with Y-39983 HCl, the cell-surface expression of BST-2 was detected by flow cytometry. *, *p* < 0.05; **, *p* < 0.01; ***, *p* < 0.001; ****, *p* < 0.0001 vs. Vpu group. Values presented are the mean ± SD of three replicates. All experiments were performed thrice.

To explore whether the disruption of Vpu and BST-2 interaction could reverse the Vpu-mediated downregulation of BST-2, HEK293T cells were transfected with CLV/NSB vectors and treated with Y-39983 HCl, brazilin, tangeretin, or AS1517499 at 10 μmol/L for 36 h. The expression of total cellular BST-2 was imaged and measured using a high-content imaging system, which showed that Vpu induced a remarkable downregulation of BST-2 (by 55%) and that this effect was reversed by the addition of Y-39983 HCl and AS1517499 (Figure 3A). Similar trends were observed in Western blotting (Figure 3B). Since HEK293T cells were known as BST-2 negative cells, we also examined the endogenous BST-2 levels in Hela cells after transfection with Vpu vector (CLV). Consistent with the findings in HEK293T cells, the results of immunofluorescence analysis (Figure 3C) and Western blotting (Figure 3D) in Hela cells showed that Y-39983 HCl and AS1517499 restored the total cellular level of endogenous BST-2 in the presence of Vpu.

Furthermore, we analyzed cell surface BST-2 levels by flow cytometry and found that only Y-39983 HCl upregulated the surface expression of BST-2 in the presence of Vpu (Figure 3E), whereas others had no effect on the surface BST-2 levels (Figure S2). These data suggest a protective role of Y-39983 HCl against Vpu induced degradation of total cellular BST-2 as well as the cell surface BST-2.

### 2.4. Antiviral Activity of Y-39983 HCl

**Figure 4:**
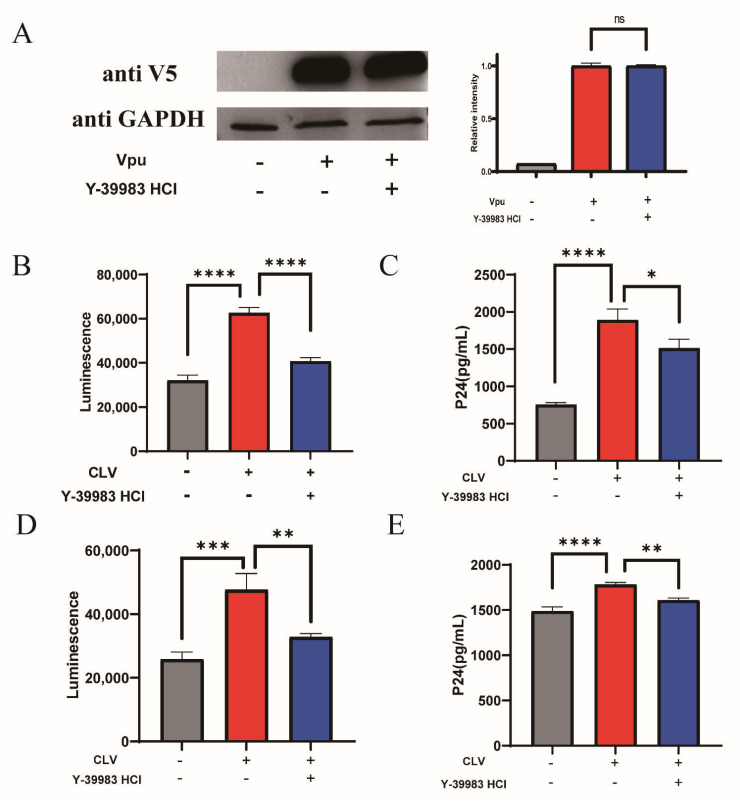
Y-39983 HCl reduced HIV-1 particle release. HEK293T cells were co-transfected with CLV-NSB plasmid or empty LgBiT vector (CLG)-NSB. Four hours after transfection, the cells were co-transfected with HIV Env expression plasmid pREJO4541.67 and backbone vector pSG3∆env in the presence of Y-39983 HCl. After 2 days, cells were lysed to detect Vpu expression by Western blotting (**A**). Env-pseudotyped virions were collected from the supernatant and used to infect TZM-bl cells. Two days after infection, the cells were lysed to evaluate luciferase activity using Bright-Glo Luciferase Assay System (**B**) and the supernatant was collected for p24 detection using ELISA assay (**C**). (**D**) HeLa cells were transfected with plasmid CLV or CLG. After 4 h, cells were co-transfected with HIV Env expression plasmid pREJO4541.67 and backbone vector pSG3∆env in the presence of Y-39983 HCl. Virions were collected from the supernatant and quantitated using TZM-bl assay. The luciferase activity was measured using Bright-Glo Luciferase Assay System. The supernatant of TZM-bl culture were collected for HIV-1 p24 detection using ELISA assay (**E**). ns, Not Significant; *, *p* < 0.05; **, *p* < 0.01; ***, *p* < 0.001; ****, *p* < 0.0001 vs. Vpu group. Values presented are the mean ± SD. All experiments were repeated thrice.

We evaluated the antiviral activity of Y-39983 HCl on the production of new pseudotyped viral particles. HEK293T cells were co-transfected with the Vpu vector (CLV), BST-2 vector (NSB) and proviral vectors with defective *vpu* gene in the presence of Y-39983 HCl. After transfection, the Vpu expression was detected by Western blotting and showed an overexpression of Vpu in transfected HEK293T cells, and Y-39983 HCl did not affect the expression of Vpu protein (Figure 4A), indicating that the inhibition effect of Y-39983 HCl on Vpu-BST-2 interaction was not mediated by affecting Vpu expression. Next, the envelope (Env)-pseudotyped virions were collected from the supernatant and tested using TZM-bl pseudovirus assay. TZM-bl is a Hela-derived cell line that expresses CD4, CCR5/CXCR5 to allow virus entry and contains a luciferase reporter gene under the control of an HIV Tat-responsive reporter. Over the years, the TZM-bl assay has been standardized as a classical method to quantify the infectious viral particle. The data showed a significant increase in the infectivity of new virions after Vpu overexpression, and the effect of Vpu was antagonized by Y-39983 HCl (Figure 4B). The virus stock was then titrated in TZM-bl cells to determine the 50% Tissue Culture Infective Dose (TCID_50_) and we found that overexpression of Vpu promoted a higher production of new virions (TCID_50_ 40,500/mL) compared to the control (TCID_50_ 23,380/mL), and treatment with Y-39983 HCl decreased virion release (TCID_50_ 28,080/mL). The inhibition of virion production by Y-39983 HCl was further confirmed by the measurement of HIV-1 P24 Gag protein using enzyme-linked immunosorbent assay (ELISA; Figure 4C). In addition, we tested AS1517499 on virus production and found no antiviral activity, despite the fact that AS1517499 restored total cellular BST-2 level (Figure S3).

We also used CLV vector transfected Hela cells to produce envelope (Env)-pseudotyped virions and quantify virus via the TZM-bl luciferase reporter assay. We found that Y-39983 HCl significantly reduced the increased infectivity of new virions induced by Vpu (Figure 4D). The TCID_50_ assay also revealed that Vpu increased the production of new virions (TCID_50_ 17,523/mL) compared to the control (TCID_50_ 7015/mL), and the effect of Vpu was antagonized by Y-39983 HCl (TCID_50_ 8424/mL). We obtained a similar result from HIV capsid P24 protein ELISA assay (Figure 4E).

## 3. Discussion

In the present study, we developed a NanoBiT-based high-throughput screening assay to identify inhibitors of Vpu-BST-2 interactions. Out of 1177 compounds, we successfully identified four that blocked Vpu-BST-2 binding. Our data showed that one compound, Y-39983 HCl, significantly inhibited Vpu-mediated degradation of total cellular and cell-surface BST-2. Moreover, Y-39983 HCl reduced the production of newly synthesized viral particles via BST-2 restoration.

NanoBiT technology was used in our work to set up a real-time assay for monitoring Vpu-BST-2 interactions in HEK293T cells. The NanoBiT assay is based on structural complementation between the LgBiT, (156 amino acids) and SmBiT (11 amino acids) of NLuc [25]. The fragments by themselves are not active, but when protein pairs of interest interact with each other, both fragments are brought into close proximity and reconstitute the intact NLuc that can be quantitatively assessed. The assay was a powerful tool in characterizing intercellular protein–protein interactions in live cells [25,26]. To optimize it for the Vpu-BST-2 interaction assay, we produced eight recombinant protein pairs to investigate whether the position and size of tags had an impact on luciferase signal. Numerous works [19,27,28,29,30] have identified that Vpu and BST-2 directly interact with each other via the transmembrane domain. Our data revealed that placing a tag at the N- and C-terminus of BST-2 and Vpu proteins, respectively, did not interfere with protein–protein binding, whereas a LgBiT tag fused to the C-terminus of BST-2 (CLB) showed a clear reduction in Vpu binding capacity, probably due to a change in protein localization. Protein pairs (CLV-NSB) that produced the highest luciferase signals were selected for subsequent experiments.

In previous studies, several compounds have been reported to protect BST-2 from Vpu-mediated degradation [23,24,31]; however, their mechanism of action is unclear. Here, using the NanoBiT-based Vpu-BST-2 interaction assay, we screened a total of 1177 small molecules and identified four compounds (Y-39983 HCl, brazilin, tangeretin, and AS1517499) with inhibitory activity against Vpu-BST-2 interaction. Y-39983 HCl is a selective Rho-associated protein kinase (ROCK) inhibitor [32,33], As1517499 is a signal transducer and activator of transcription 6 inhibitor, and tangeretin and brazilin are natural products with multiple functions. None of them had previously been reported to have anti-HIV activity. We also calculated the IC_50_ and EC_50_ to monitor the safety and efficacy of the compounds and found that all compounds exhibited dose-dependent activity and low cytotoxicity.

Next, we investigated whether inhibitors could inhibit the degradation of BST-2 induced by Vpu. As is known, HEK293T cells are BST-2 negative cells and Hela cells constitutively expressed BST-2 protein. We used both cell lines in our study and the results were consistent with those of previous studies [19,29], which showed that Vpu induced downregulation of cell-surface and total cellular expression of BST-2. It has been noted that Vpu-mediated BST-2 degradation varies depending on the cell type [28]. Vpu was shown to downregulate BST-2 at the cell surface as well as remove it from viral budding sites and prevent the recycling of BST-2 or *de novo*-synthesized BST-2 to the plasma membrane [34,35]. Although the degradation mechanisms remain controversial, the importance of Vpu-mediated surface downregulation of BST-2 in viral egress is well-defined [36]. In our study, with the addition of Y-39983 HCl and AS1517499, the total cellular levels of BST-2 were restored despite the presence of Vpu. However, only Y-39983 HCl upregulated the cell-surface expression of BST-2. Therefore, Y-39983 HCl may have the ability to prevent virus release via BST-2 restoration on the cell surface and was selected for further evaluation.

Our results also suggest that the infectivity of HIV-1 virus can be inhibited by Y-39983 HCl. HIV-1 Env-pseudotyped viruses were produced by transfection of HeLa and HEK293T cells with proviral DNA constructs (with defects in the *vpu* gene) and assessed using a TZM-bl assay. This assay is a quantitative method of measuring infectious viruses by a Tat-responsive Luc reporter and is widely used as a standardized HIV-1 neutralization assay [37,38]. Viruses were titrated on TZM-bl cells to calculate the viral titer (TCID_50_/mL) [39] and HIV-1 P24 antigen capture assay, a long-established method for detecting HIV-1 virus replication, was also used for virus quantitation [40,41]. Using TZM-bl cells, we found that overexpression of Vpu led to an increase in the infectivity of the released virus and the data are consistent with previous reports that demonstrated that Vpu promoted viral release by antagonizing BST-2 [14,31,42]. With the addition of Y-39983 HCl, virus production was significantly inhibited, indicating that disruption of Vpu-BST-2 interaction and protection of BST-2 expression on cell surface contributed to the antiviral activity of Y-39983 HCl.

## 4. Materials and Methods

### 4.1. Cell Culture and Transfection

HEK293T and HeLa cells were obtained from the American Type Culture Collection (ATCC, Rockville, MD, USA), and TZM-bl cells were obtained from the National Institutes of Health [NIH] AIDS Research and Reference Reagent Program. TZM-bl (JC, 53bl-13) cell line is a genetically modified HeLa cell line that expresses CD4 and CCR5/CXCR4 and contains the firefly luciferase and the LacZ reporter gene under the control of a Tat-responsive reporter for the detection of Tat-dependent luciferase and β-galactosidase activities after Env-pseudo typed viral infection. All cells were cultured in Dulbecco’s modified Eagle medium (Gibco, Grand Island, NY, USA) supplemented with 10% fetal bovine serum (FBS) (Gibco) and penicillin-streptomycin (Gibco). HEK293T and TZM-bl cells were transfected using Lipofectamine™ 3000 Transfection Reagent (Invitrogen, Carlsbad, CA, USA) according to the manufacturer’s instructions.

### 4.2. Plasmid and Compound Library

The cloning vectors used to create LgBiT and SmBiT protein fusions were provided in the Nano-Glo^®^ Live Cell Assay System (Promega, Madison, WI, USA). The *vpu* and *BST-2* genes were subcloned into pBiT1.1-C [TK/LgBiT], pBiT2.1-C [TK/SmBiT], pBiT1.1-N [TK/LgBiT], and pBiT2.1-N [TK/SmBiT] vectors at the *XhoI* and *NheI* sites, respectively, to generate the fusion protein expression vector. A library of 1117 bioactive compounds was obtained from Selleck Chemicals (L4100 and L2000; Houston, TX, USA). Y-39983 HCl (S7935), brazilin (S9428), tangeretin (S2363), and AS1517499 (S8685) were also obtained from Selleck Chemicals. The structure of 4 compounds is shown in Figure S1.

### 4.3. Western Blotting and Antibodies

HEK293T and HeLa cells were lysed on ice for 15 min in 100 μL radioimmunoprecipitation assay lysis buffer (Solarbio, Beijing, China) supplemented with a protease inhibitor cocktail (Promega). The protein concentration of lysates was determined using a bicinchoninic acid protein assay kit (Solarbio), and equal amounts of cellular proteins were suspended in NuPAGE LDS sample buffer (Invitrogen) and NuPAGE™ sample reducing buffer (Invitrogen), followed by boiling for 5 min at 95 °C. Proteins were then separated on 12% Mini-PROTEAN TGX gels (Bio-Rad, USA) and transferred onto polyvinylidene difluoride membranes. The membranes were then blocked with 5% non-fat milk in phosphate-buffered saline (PBS) containing 0.01% Tween-20 and blotted with rabbit anti-human BST-2 (Abcam, UK) or rabbit anti-human β-actin antibodies (Abcam) overnight at 4 °C. The express of V5 tagged Vpu protein was detected by mouse anti-V5 antibody (Thermo). The membranes were further incubated with horseradish peroxidase-labeled goat anti-rabbit secondary antibodies or goat anti-mouse secondary antibodies and detected using enhanced chemiluminescence. The protein bands were imaged using a Tanon 5200 multi-imaging system (Tanon Science& Technology Co., Ltd., Shanghai, China).

### 4.4. Flow Cytometry

For cell-surface staining, HEK293T cells were collected and stained with PE anti-human CD317 (BioLegend, CA, USA) at room temperature for 30 min. Cells were then washed and resuspended in PBS + 1% FBS for FACS analysis using a BD FACS Calibur^TM^ flow cytometer (BD Bioscience, San Diego, CA, USA). The data were analyzed using the FlowJo software V10 (Tree Star, Ashland, OR, USA).

### 4.5. NanoBiT Assay

HEK293T cells were plated in 96-well plates at a concentration of 1 × 10^5^ cells/mL and co-transfected with the plasmids encoding the Vpu and BST-2 fusion proteins at a ratio of 1:1. The empty vector (HaloTag with SmBiT) was used as a negative control. At 12 h post-transfection, the cells were treated with bioactive compounds (L2000 and L4100; Selleck Chemicals) at a final concentration of 10 μmol/L and incubated for 24 h. Then, Nano-Glo Live Cell Substrate (N2012; Promega) was added and luminescence was measured using an EnSpire Multilabel Plate Reader 2300 (PerkinElmer, Waltham, MA, USA). The inhibition rate on Vpu-BST-2 binding was calculated as, inhibition rate (%) = (1 − experimental OD value/control OD value) × 100%. A heat map of the inhibition rate of all compounds was generated using GraphPad Prism 8 software (GraphPad Software Inc., San Diego, CA, USA).

### 4.6. Virus Release and Infectivity Analysis

HEK293T and HeLa cells were seeded in 6-well plates. HEK293T cells were transfected with NSB and CLV plasmids containing the *bst-2* and *vpu* genes. The empty large BiT vector (CLG) was used as a negative control. HeLa cells were transfected with CLV or CLG plasmids. Four hours after transfection, the cells were co-transfected with HIV Env expression plasmid pREJO4541.67 (NIH#11035) and backbone vector pSG3∆env (NIH#11051; NIH AIDS Research and Reference Reagent Program) in the presence of Y-39983 HCl. After 2 d, recombinant viruses were collected from the supernatants. Then, TZM-bl cells were seeded in a 96-well plate in medium containing 10 μg/mL DEAE-dextran (Sigma) and infected with the Env-pseudotyped virus. Two days after infection, the supernatant was collected to quantify P24 level by HIV-1 p24/Capsid Protein p24 ELISA Kit (SinoBiological) following the manufacturer’s instruction. Additionally, the cells were lysed to evaluate luciferase activity using the Bright-Glo Luciferase Assay System (Promega). To calculate the 50% tissue culture infective dose (TCID_50_) of the Env-pseudotyped virus, TZM-bl cell were infected with serial 3-fold dilutions of the Env-pseudotyped virus (starting at 1: 10 dilutions). Two days after infection, cells were lysed to evaluate luciferase activity using the Bright-Glo Luciferase Assay System, TCID_50_ was calculated using the Reed-Muench method [43,44].

### 4.7. Immunofluorescence

HEK293T and HeLa cells were seeded in 6-well plates. HEK293T cells were transfected with NSB and CLV plasmids containing the *bst-2* and *vpu* gene. The empty large BiT vector (CLG) was used as a negative control. HeLa cells were transfected with CLV or CLG plasmids. Twenty-four hours after transfection, the cells were fixed with 4% paraformaldehyde and permeabilized with 0.5% Triton X-100. After blocking with normal goat serum (Solarbio), the cells were stained with rabbit anti-human BST-2 antibody for 2 h at 37 °C and then incubated with Alexa Fluor 594-conjugated goat anti-rabbit secondary antibody. The cells were then incubated with DAPI solution (Solarbio) for 10 min at room temperature. Immunofluorescence was imaged using the Operetta high-content imaging system (PerkinElmer) and quantitatively analyzed using the Harmony software (PerkinElmer). The mean fluorescence was calculated as follows: fluorescence per nucleus = fluorescence per well/cell number (DAPI positive cells).

### 4.8. Cell Viability

Cell viability was assessed using Cell Counting Kit-8 (CCK-8; Dojindo Molecular Technologies Inc., Tokyo, Japan). Briefly, HEK293T cells were treated with a serial dilution of Y-39983 HCl, brazilin, tangeretin, or AS1517499 ranging from 0.4 to 50 μmol/L. Forty-eight hours after treatment, the cells were incubated with CCK-8 solution and absorbance was measured at 450 nm using an EnSpire Multilabel Plate Reader 2300 (PerkinElmer), and the relative cell viability was calculated as follows: cell viability (%) = experimental OD value/control OD value × 100%.

### 4.9. Statistical Analysis

Data are presented as the mean ± standard deviation, as indicated in the figure legends. Statistical analysis was performed by one-way analysis of variance using GraphPad Prism 8 software (GraphPad Software Inc., San Diego, CA, USA). Differences between groups were considered significant at *p* < 0.05.

## 5. Conclusions

Using NanoBiT technology, we developed a high-throughput screening assay to monitor Vpu-BST-2 interactions in live cells. We identified four compounds with inhibitory activity against the Vpu-BST-2 interaction. We further revealed that one compound, Y-39983 HCl restored BST-2 expression on the cell surface and inhibited viral particle production. Therefore, our study suggests a new application of NanoBiT technology in the development of novel antiviral drugs.

## Supplementary Materials

The following are available online at https://www.mdpi.com/article/10.3390/ijms22179308/s1.
