# Supplementary material for: High-Throughput NanoBiT-Based Screening for Inhibitors of HIV-1 Vpu and Host BST-2 Protein Interaction
Source: Int J Mol Sci. 2021 Aug 27;22(17):9308. doi: 10.3390/ijms22179308 (PMC8431494; doi:10.3390/ijms22179308)
Supplement: Supplementary file 1 [file ijms-22-09308-s001.zip › ijms-1323972-supple-revised/ijms-1323972-Supplementary.pdf]

International Journal of
Molecular Sciences

MDPI

Supplementary Materials for

High-Throughput NanoBiT-Based Screening for Inhibitors of HIV-1 Vpu and host BST-2 Protein Interaction

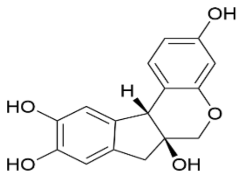

Brazilin

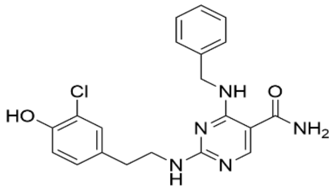

AS1517499

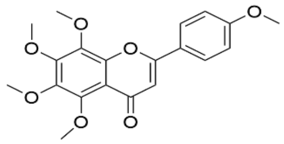

AS1517499

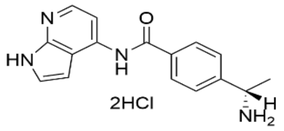

Y-39983 HCl

Supplemental Figure S1. Chemical structure of the four compounds

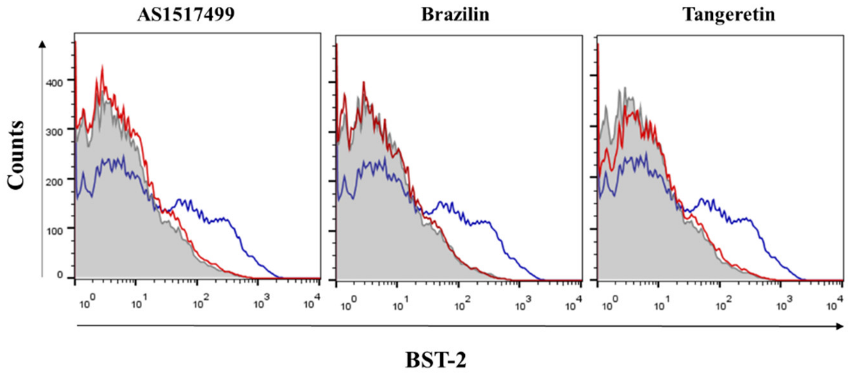

Supplemental Figure S2. The cell-surface expression of BST-2 in the presence of AS1517499, Brazilin and Tangeretin. HEK293T cells were co-transfected with plasmids CLV (Vpu with C-terminal LgBiT) and NSB (BST-2 with N-terminal SmBiT), and treated with brazilin, tangeretin, or AS1517499 (10 μmol/L) for 36 h. The cell-surface expression of BST-2 was detected by flow cytometry. The blue line present control group, grey line present Vpu group and red line present compounds treated group.

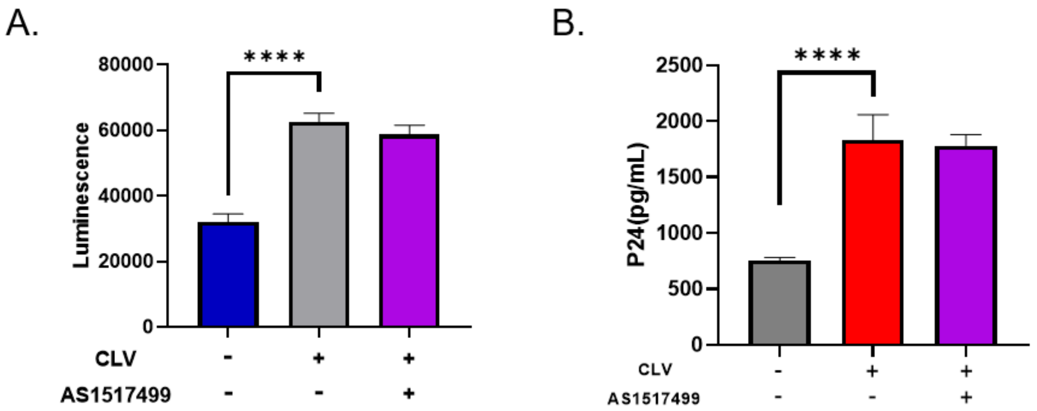

Supplemental Figure S3. Effect of AS1517499 on viral release from Vpu mediated BST-2 down regulated cells. (A) HEK293T cells were transfected with NSB-CLV plasmids or NSB and empty LgBiT vector (CLG). Four hours after transfection, the cells were co-transfected with HIV Env expression plasmid pREJO4541.67 and backbone vector pSG3Δenv in the presence of AS1517499. Env-pseudotyped virions were collected from the supernatant and used to infect TZM-bl cells. Two days after infection, the cells were lysed to evaluate luciferase activity using Bright-Glo Luciferase Assay System (B) and the supernatant was collected for HIV-1 capsid p24 protein ELISA to confirm the inhibition on virions production by AS1517499.

Supplemental Table S1. Cell viability, inhibition rate, and therapeutic index of the four compounds

Compounds	EC50[a]	CC50[b]	TI[c]
Brazilin	5.62	25.18	4.5
Tangeretin	3.36	>50	>14.8
AS1517499	0.55	25.83	47
Y-39983 HCl	0.55	>50	>91

[a]EC50 refers to the concentration of the compound sufficient to reduce the inhibition rate of Vpu-BST-2 by 50% .

[b]CC50 refers to the concentration of the compound sufficient to reduce cell viability by 50% .

[c]Therapeutic index (TI) is calculated by CC50/EC50.

Supplemental Table S2. A library of 1117 bioactive compounds

S1013	S2170	S1089	S2238	S1153	S2306	S1210	S2333	S1290	S2351
Bortezomib (PS-341)	Givinostat (ITF2357)	Refametinib (RDEA119, Bay 86-9766)	Palomid 529 (P529)	Roscovitine (Seliciclib,CYC 202)	Gynostemma Extract	Methotrexate	Nobiletin	Celastrol	Salicin
S1623	S2729	S1639	S2806	S1679	S2863	S1706	S2913	S1743	S3027
Acetylcysteine	SB415286	Amprenavir	CEP-33779	Flurbiprofen	ML130 (Nodinitib-1)	Lamivudine	BAY 11-7082	NEXIUM (esomeprazole magnesium)	Fenoprofen calcium hydrate
S1039	S2173	S1102	S2250	S1157	S2308	S1233	S2336	S1291	S2354
Rapamycin (Sirolimus)	Telotristat Etiprate (LX 1606 Hippurate)	U0126-EtOH	(-)-Epigallocatechin Gallate	Delanzomib (CEP-18770)	Hesperetin	2-Methoxyestradiol (2-MeOE2)	Orotic acid (6-Carboxyuracil)	Cetirizine DiHCl	Sclareol
S1626	S2773	S1643	S2813	S1681	S2864	S1713	S2918	S1761	S3032
Naproxen Sodium	SB705498	Ursodiol	Ciproxifan Maleate	Mesalamine	IMD 0354	Piroxicam	HC-030031	Suprofen	Bindarit
S1049	S2180	S1110	S2269	S1177	S2314	S1247	S2337	S1299	S2357
Y-27632 2HCl	Ixazomib (MLN2238)	Varespladib (LY315920)	Baicalin	PD98059	Kaempferol	Leflunomide	Osthole	Floxuridine	Silibinin
S1627	S2785	S1645	S2822	S1688	S2874	S1718	S2928	S1771	S3037
Nitazoxanide	A-803467	Ketoprofen	OC000459	Betamethasone Dipropionate	Camostat Mesilate	Adefovir Dipivoxil	TAK-715	Chlorprothixene	Bepotastine Besilate
S1064	S2181	S1115	S2270	S1183	S2317	S1252	S2338	S1305	S2358
Masitinib (AB1010)	Ixazomib Citrate (MLN9708)	Odanacatib (MK-0822)	Bergenin	Danoprevir (ITMN-191)	L-(+)-Rhamnose Monohydrate	Entecavir Hydrate	Oxymatrine	Mercaptopurine (6-MP)	Silymarin
S1628	S2787	S1646	S2823	S1689	S2902	S1721	S3001	S1774	S3051
Triamcinolone Acetonide	Laquinimod	Ketorolac	Tideglusib	Meprednisone	S-Ruxolitinib (INCB018424)	Azathioprine	Clevudine	Thioguanine	Bosentan Hydrate
S1072	S2194	S1120	S2276	S1185	S2318	S1255	S2339	S1322	S2359
ZSTK474	R406	Everolimus (RAD001)	Bilobalide	Ritonavir	Lappaconitine	Nepafenac	Paeonol	Dexamethasone (DHAP)	Sinomenine
S1630	S2789	S1651	S2824	S1690	S2903	S1733	S3008	S1786	S3052
Allopurinol	Tofacitinib (CP-690550,Tasocitinib)	Telbivudine	TPCA-1	Betamethasone Valerate	Lumiracoxib	Methylprednisolone	Zaltoprofen	Verteporfin	Rupatadine Fumarate
S1075	S2226	S1129	S2277	S1193	S2319	S1261	S2347	S1328	S2360
SB216763	Idelalisib (CAL-101, GS-1101)	SRT1720 HCl	Caffeic Acid	Thalidomide	Limonin	Celecoxib	Quercetin Dihydrate	Etodolac	Solanesol (Nonaisoprenol)
S1631	S2791	S1653	S2832	S1696	S2905	S1735	S3017	S1790	S3062
Allopurinol Sodium	Sotrastaurin	Tretinoin	Epiandrosterone	Hydrocortisone	JNJ-7777120	Mesna	Aspirin	Rifaximin	Diclofenac Potassium
S1076	S2228	S1137	S2286	S1199	S2325	S1276	S2349	S1340	S2363
SB203580	Belnacasan (VX-765)	Malotilate	Cyclosporin A	Cladribine	Morin Hydrate	Adapalene	Rutaecarpine	Gatifloxacin	Tangeretin
S1633	S2794	S1654	S2851	S1701	S2907	S1737	S3023	S1792	S3063
Zafirlukast	Sofosbuvir (PSI-7977, GS-7977)	Phenylbutazone	Baricitinib (LY3009104, INCB028050)	Desonide	Pirfenidone	Prednisolone	Bufexamac	Simvastatin	Diclofenac Diethylamine
S1077	S2233	S1152	S2302	S1204	S2332	S1286	S2350	S1343	S2365
SB202190 (FHPI)	Esomeprazole sodium	PLX-4720	Glycyrrhizin (Glycyrrhizic Acid)	Melatonin	Neohesperidin	Budesonide	Rutin	Ginkgolide B	Tanshinone IIA
S1638	S2797	S1669	S2853	S1704	S2910	S1740	S3026	S1801	S3078
S1352	S2383	S1385	S2413	S1439	S2512	S1482	S2570	S1567	S2608
TG100-115	Gastrodin	Mosapride Citrate	Geniposidic acid	Tranilast	Tenoxicam	Daclatasvir (BMS-790052)	Prednisolone Acetate	Pomalidomide	Fluocinonide
S1802	S3083	S1866	S3137	S1987	S3610	S2037	S3654	S2074	S3687
AICAR (Acadesine)	Indacaterol Maleate	Diphenhydramine HCl	Sodium salicylate	Mometasone furoate	Cordycepin	Candesartan Cilexetil	Tauroursodeoxycholic Acid (TUDCA)	Mecarbinate	E-Cardamoni
S1354	S2387	S1389	S2425	S1443	S2517	S1500	S2584	S1569	S2625
Lansoprazole	Lappaconite HBr	Omeprazole	Apocynin	Zileuton	Maprotiline HCl	Betamethasone	Clobetasol propionate	Tazarotene	Fostamatinib (R788)
S1816	S3104	S1880	S3176	S1992	S3612	S2040	S3666	S2075	S3694
Chlorpheniramine Maleate	Moguisteine	Roxatidine Acetate HCl	Betahistine 2HCl	Fluticasone propionate	Rosmarinic acid	Nimesulide	Ilaprazole	Rosiglitazone HCl	Glucosamine hydrochloride
S1358	S2390	S1396	S2439	S1455	S2547	S1501	S2585	S1574	S2627
Loratadine	Polydatin	Resveratrol	Guanosine	Cilomilast	Tiotropium Bromide hydrate	Mycophenolate Mofetil	Brompheniramine hydrogen maleate	Doramapimod (BIRB 796)	Tubastatin A HCl
S1823	S3105	S1888	S3186	S2003	S3613	S2046	S3668	S2078	S3698
Fenoprofen Calcium	Nadifloxacin	Deflazacort	Azatadine dimaleate	Maraviroc	Scoparone	Pioglitazone HCl	Thymopentin	Famotidine	Nortriptyline hydrochloride
S1368	S2391	S1400	S2470	S1457	S2552	S1508	S2586	S1576	S2630
Acitretin	Quercetin	Tenofovir Disoproxil Fumarate	Fluocinolone Acetonide	Atazanavir Sulfate	Azelastine HCl	Alprostadil	Dimethyl Fumarate	Sulfasalazine	GW3965 HCl
S1825	S3106	S1890	S3208	S2007	S3615	S2060	S3675	S2083	S3711
Erdosteine	Pidotimod	Nizatidine	Fexofenadine HCl	Sulindac	Dehydrocostus Lactone	Bromhexine HCl	Umbelliferone	Procaterol HCl	Carbasalate Calcium
S1378	S2394	S1401	S2494	S1458	S2554	S1514	S2590	S1608	S2681
Ruxolitinib (INCB018424)	Naringenin	Tenofovir	Olopatadine HCl	VX-745	Daphnetin	Cyclosporine	Pioglitazone	Pyridostigmine Bromide	AS-604850
S1829	S3120	S1895	S3603	S2015	S3616	S2062	S3679	S2101	S3714
Pranlukast	Doxepin HCl	Dipyridamole	Betulinic acid	Suplatast Tosylate	Asiaticoside	Tiopronin	Flavanone	Gabexate Mesylate	Lifitegrast

S1379	S2399	S1407	S2496	S1460	S2555	S1533	S2602	S1609	S2694
Isotretinoin	Dihydromyricetin	Bimatoprost	Ozagrel	SP600125	Clarithromycin	R406 (free base)	Acemetacin	Methimazole	Turofexorate Isopropyl (XL335)
S1845	S3124	S1933	S3604	S2020	S3622	S2065	S3680	S2108	S3720
Cimetidine	Dexamethasone Acetate	Triamcinolone	Triptolide (PG490)	Formoterol Hemifumarate	Diammonium Glycyrrhizinate	Lafutidine	NLRP3 Inflammasome Inhibitor I	Flunixin Meglumin	Elafibranor
S1380	S2407	S1430	S2502	S1467	S2556	S1538	S2603	S1620	S2700
Lopinavir	Curcumol	Rolipram	Quinine HCl Dihydrate	Doxercalciferol	Rosiglitazone	Telaprevir (VX-950)	Tioxolone	Darunavir Ethanolate	KX2-391
S1847	S3130	S1952	S3605	S2024	S3632	S2067	S3684	S2127	S3724
Clemastine Fumarate	Biotin (Vitamin B7)	Methoxsalen	Borneol	Ketotifen Fumarate	Trichloroisocyanuric acid	Ozagrel HCl	Methacholine chloride	S-(+)-Rolipram	Velpatasvir
S1382	S2410	S1435	S2505	S1480	S2559	S1547	S2605	S1622	S2726
Mianserin HCl	Paeoniflorin	Tamsulosin	Rosiglitazone maleate	Lomibuvir (VX-222, VCH-222)	Cortisone acetate	Febuxostat	Idebenone	Prednisone	PH-797804
S1848	S3133	S1973	S3608	S2032	S3648	S2071	S3685	S2131	S3726
Curcumin	Sulfamethazine	Cyclocytidine HCl	Demethylzeylasteral (T-96)	Rebamipide	Amlexanox	Prulifloxacin (NM441, AF 3013)	4-Biphenylacetic acid	Roflumilast	Selexipag
S3727	S4368	S3764	S4525	S3780	S4577	S3807	S4625	S3827	S4690
Vilanterol Trifenate	Carbenoxolone Sodium	Isoferulic Acid	Ethylparaben	Secoisolariciresinol diglucoside	Terpin hydrate	Dehydroandrographolide	Alcaftadine	Royal jelly acid	Escin
S3934	S5010	S3967	S5043	S4007	S5105	S4049	S5151	S4095	S5215
Acetyl Resveratrol	Indometacin Sodium	Flavone	Benorylate	Pentamidine isethionate	Proanthocyanidins	Valdecoxib	Gypenoside	Difluprednate	Abacavir
S3728	S4398	S3765	S4526	S3783	S4580	S3808	S4630	S3829	S4695
Grazoprevir	Isoxicam	Picroside II	Fenbufen	Echinacoside	Hydroquinone	Mangiferin	Diazoxide	Isoalantolactone	D panthenol
S3937	S5014	S3968	S5044	S4011	S5109	S4051	S5157	S4098	S5227
Pterostilbene	Crisaborole (AN2728)	Histamine	Isoxepac	Ampiroxicam	Ademetionine	Nabumetone	Thymol	Halcinonide	Erythromycin thiocyanate
S3733	S4502	S3766	S4529	S3784	S4586	S3810	S4646	S3834	S4696
Boceprevir	Eltrombopag	Tanshinone IIA sulfonate (sodium)	Idramantone	Obacunone	4-Chloro-DL-phenylalanine	Scutellarin	Ciclesonide	Astaxanthin	CarbinoxaMine Maleate
S3938	S5015	S3969	S5046	S4012	S5110	S4074	S5168	S4099	S5233
Bisdemethoxycurcumin (BDMC)	Simeprevir	Veratric acid	Clonixin	Desloratadine	Safflower Yellow	Sodium Nitrite	Anthraquinone	Dexlansoprazole	Fenretinide
S3735	S4504	S3774	S4535	S3787	S4589	S3813	S4651	S3835	S4699
Umeclidinium bromide	6-Mercaptopurine (6-MP) Monohydrate	Dehydroandrographolide Succinate Potasium Salt	Methylene Blue	Picroside I	Amodiaquine dihydrochloride dihydrate	Bakuchiol	Etoricoxib	Loganin	Etretinate
S3941	S5016	S3975	S5049	S4022	S5113	S4078	S5177	S4107	S5234
Pinocembrin	Isoprinosine	Protocatechuic acid	Thiocolchicoside	Probenecid	Propyl gallate	Mefenamic Acid	Naproxen	Clofazimine	Nintedanib Ethanesulfonate Salt
S3739	S4509	S3775	S4539	S3788	S4590	S3817	S4662	S3836	S4706
Calcipotriene	4-Aminoantipyrine	Ligustrazine hydrochloride	Salicylic acid	Carvacrol	Dithranol	Harmine hydrochloride	Atazanavir	6-Gingerol	Eugenol
S3942	S5020	S3982	S5058	S4026	S5121	S4083	S5179	S4118	S5243
Cardamonin	Tilorone dihydrochloride	Batyl alcohol	Revaprazan Hydrochloride	Hydroxyzine 2HCl	Phytol	Vitamin A Acetate	Ropivacaine Mesilate	Histamine 2HCl	Ruxolitinib Phosphate
S3745	S4511	S3776	S4548	S3790	S4603	S3818	S4666	S3837	S4708
Balsalazide disodium	6-Benzylaminopurine	Sophoricoside	Aminoguanidine hydrochloride	Methyl gallate	Gallic acid	Tectoridin	Sivelestat sodium tetrahydrate	Echinocystic acid	Palmitoylethanolamide
S3945	S5023	S3983	S5062	S4041	S5122	S4088	S5200	S4128	S5246
L-Cycloserine	Nadolol	Caryophyllene oxide	Daclatasvir Digydrochloride	Olsalazine Sodium	Abietic Acid	Flumethasone	Diaveridine	Troxipide	Entecavir
S3758	S4515	S3777	S4554	S3799	S4604	S3824	S4682	S3838	S4709
Sinomenine hydrochloride	Ademetionine disulfate tosylate	Gentiopicroside	Bucetin	Gentisic acid	Levofloxacin hydrate	Quercitrin	Loxoprofen	Carnosic acid	Latanoprost
S3951	S5028	S3990	S5083	S4045	S5138	S4089	S5210	S4131	S5250
Tannic acid	4-Aminopyridine	Trans-Anethole	Lentinan	Pheniramine Maleate	Citral	Halobetasol Propionate	Sulfamethazine Sodium Salt	Levodropropizine	Darunavir
S3761	S4524	S3778	S4555	S3804	S4609	S3826	S4686	S3840	S4711
Eucalyptol	2-Ethoxybenzamide	Patchouli alcohol	Carsalam	Alpha-Mangostin	Diflunisal	Hydroxytyrosol	Vitamin E	Baohuoside I	Esculetin
S3956	S5039	S3997	S5103	S4048	S5141	S4090	S5211	S4139	S5258
Tetramethylpyrazine	Actarit	Oxaceprol	lutein	Carbimazole	Pyrithioxin	Fenspiride HCl	4-Aminosalicylic acid	Cyclizine 2HCl	Revefenacin
S3841	S4716	S3868	S4759	S3880	S4839	S3899	S4935	S3925	S4986
Eleutheroside B	Evans Blue	Harmine	p-Coumaric Acid	Schisantherin A	Mosapride	Hederagenin	Asunaprevir	(-)-Epicatechin gallate	Latamoxef sodium
S4149	S5261	S4177	S5322	S4230	S5406	S4282	S5459	S4330	S5486
Amfenac Sodium Monohydrate	Ufenamate	Uracil	Sodium gualenate	Oxaprozin	Sultamicillin Tosylate	Nelfinavir Mesylate	Aucubin	Isoetharine Mesylate	Fluorometholone
S3846	S4722	S3869	S4761	S3881	S4845	S3901	S4938	S3926	S4990
Eupatilin	(+)-Catechin	Methyl protocatechuate	Thymoquinone	Scopoletin	Rabeprazole	Astragaloside IV	Cedrol	Forsythin	TBHQ
S4152	S5286	S4188	S5345	S4240	S5409	S4290	S5462	S4331	S5487
Ethamsylate	Ramatroban	Sasapyrine	Nerolidol	Doxylamine Succinate	Chelidamic acid hydrate	Digoxin	5-O-Methylvisammioside	Meclocycline Sulfosalicylate	Cefoperazone sodium

S3847	S4723	S3870	S4783	S3885	S4849	S3905	S4941	S3927	S4994
Panaxatriol	(-)Epicatechin	D-Pinitol	Benzyl isothiocyanate	Pyrogallol	Levocetirizine Dihydrochloride	Iso-Steviol	Farnesol	Swertiamarin	Methylcobalamin
S4163	S5304	S4190	S5360	S4248	S5447	S4293	S5463	S4332	S5494
Doxycycline Hyclate	2-Acetylphenothiazine (ML171)	Cinchophen	Diflorasone	Bromfenac Sodium	Triprolidine Hydrochloride	Promethazine HCl	Curcurbitacin IIA	Medrysone	Salbutamol
S3848	S4728	S3871	S4789	S3890	S4852	S3906	S4952	S3928	S5001
Hydroxytyrosol Acetate	3,3'-Diindolylmethane	Muscone	5-Acetylsalicylic acid	Leonurine	Gadopentetate Dimeglumine	Astragalus polyphenols	Fumaric acid	Baccatin III	Tofacitinib (CP-690550) Citrate
S4164	S5305	S4208	S5361	S4253	S5451	S4295	S5464	S4335	S5500
Doxofylline	Bromisoval	Chromocarb	Bendazac	Epinastine HCl	Ferulic acid methyl ester	Meclofenamate Sodium	Psoralidin	Metaproterenol Sulfate	Amodiaquine hydrochloride
S3850	S4735	S3872	S4815	S3892	S4853	S3907	S4953	S3929	S5002
Glucosamine sulfate	Salvianolic acid B	Guaiacol	L-Cysteine HCl	Isopsoralen	Ecabet sodium	Bulleyaconicine A	Usnic acid	Liquiritigenin	Fingolimod (FTY720) HCl
S4165	S5307	S4210	S5399	S4260	S5452	S4296	S5465	S4341	S5501
Benzydamine HCl	PSI-6206 (RO-2433, GS-331007)	Benzocaine	Chlorprothixene hydrochloride	Tamibarotene	Sanguinarine chloride	Salmeterol Xinafoate	Morroniside	Nalmefene HCl	Hydrocortisone acetate
S3855	S4737	S3874	S4832	S3893	S4862	S3909	S4955	S3930	S5003
Ethyl ferulate	Psoralen	Curcumenol	Tolmetin	Bornyl acetate	Squalene	Catalpol	Acetosyringone	Liquiritin	Tacrolimus (FK506)
S4169	S5309	S4211	S5402	S4267	S5453	S4307	S5466	S4353	S5502
Teriflunomide	Propyphenazone (4-Isopropylantipyrine)	Montelukast Sodium	Dasabuvir(ABT-333)	Diacerein	Hyperoside	Auranofin	Saikosaponin A	Terfenadine	Ilaprazole sodium
S3859	S4746	S3877	S4835	S3895	S4890	S3922	S4966	S3931	S5004
Vanillyl Alcohol	(20S)-Protopanaxadiol	Lysionotin	Aceclofenac	Sophoridine	Bifendate	(-)-epigallocatechin	4-Methylesculetin	Ginsenoside Rd	Pimecrolimus
S4170	S5314	S4228	S5403	S4268	S5457	S4308	S5471	S4362	S5517
Coumarin	HTHQ(1-O-Hexyl-2,3,5-trimethylhydroquinone)	Fluorometholone Acetate	Ombitasvir (ABT-267)	Flufenamic acid	Curculigoside	Benzthiazide	Monotropein	Glafenine HCl	Fenamic acid
S3867	S4753	S3879	S4837	S3897	S4894	S3924	S4976	S3932	S5006
(E)-Cardamoni	Ganoderic acid A	kaempferide	Ibudilast	(-)-Arctigenin	D-Glucurone	Ginsenoside Rb1	Sulfalene(SMPZ)	Astilbin	Teprenone
S4176	S5318	S4229	S5404	S4277	S5458	S4320	S5479	S4367	S5542
Trometamol	Acacetin	Oxybuprocaine HCl	Paritaprevir (ABT-450)	Bambuterol HCl	Verbascoside	Dimaprit 2HCl	Cloperastine hydrochloride	Suxibuzone	7-Nitroindazole
S5551	S8188	S5648	S8292	S5662	S8348	S5718	S8562	S5774	S8685
6-Paradol	BFH772	Iguratimod	Selonsertib (GS-4997)	Ranitidine	BMS-935177	Acrivastine	CA-4948	CY-09	AS1517499
S7310	S9099	S7434	S9116	S7524	S9130	S7612	S9161	S7660	S9174
SF1670	Orientin	TAPI-1	Chikusetsusaponin IVa	FR 180204	Rubusoside	PX-478 2HCl	Ziyuglycoside II	Obeticholic Acid	Xanthotoxol
S5579	S8189	S5650	S8298	S5670	S8432	S5720	S8605	S5783	S8694
Chelidonic acid	BAW2881 (NVP-BAW2881)	Sodium Hyaluronate	CZ415	Chloropyramine hydrochloride	Troglitazone (CS-045)	Glecaprevir	C188-9	Montelukast	CID16020046 (CID 16020046)
S7352	S9100	S7436	S9120	S7540	S9131	S7616	S9165	S7672	S9175
Bay 11-7085	Fraxinellone	NH125	Scutellarein	SB273005	Punicalagin	CPI-169	Poncirin	Omaveloxolone (RTA-408)	Macranthoidin A
S5582	S8195	S5652	S8301	S5685	S8433	S5729	S8639	S5804	S8752
Cytarabine hydrochloride	Oclacitinib?maleate	Elbasvir	AS101	Desoximetasone	NS-398 (NS398)	Fosfosal	Diphenyleneiodonium chloride (DPI)	N-Acetylcysteine amide	leniolisib(CDZ173)
S7360	S9105	S7448	S9122	S7541	S9132	S7620	S9167	S7691	S9180
OTX015	Ginsenoside CK	CORM-3	Tectorigenin	Decernotinib (VX-509)	Harpagide	GSK1324726A (I-BET726)	Columbin	PS-1145	Tussilagone
S5619	S8236	S5654	S8305	S5686	S8484	S5737	S8640	S5834	S8761
Betahistine	MK-886 (L-663,536)	Indacaterol	TRC051384	Tryptanthrin	GSK2982772	Diclofenac Epolamine	Reparixin (Repertaxin)	p-Coumaric acid ethyl ester	Adavivint (SM04690)
S7378	S9106	S7470	S9123	S7551	S9138	S7624	S9168	S7732	S9183
AEBSF HCl	Eleutheroside E	Triapine	Eriodictyol	Piperlongumine	Picfeltarraenin IA	SD-208	Periplogenin	ISO-1	Ruscogenin
S5621	S8241	S5656	S8306	S5697	S8490	S5753	S8648	S5836	S8770
Cilastatin	Ponesimod	Diphenylpyraline hydrochloride	Leukadherin-1	Isopropamide Iodide	Tanzisertib(CC-930)	Setipiprant(ACT-129968, KYTH-105)	ACY-738	IBMX	Lanifibranor(IVA-337)
S7381	S9110	S7515	S9125	S7579	S9144	S7634	S9170	S7738	S9184
Pepstatin A	Morin	VGX-1027	Apigetrin	Ledipasvir (GS5885)	Solasonine	Cerdulatinib (PRT062070, PRT2070)	Engeletin	PRT-060318 2HCl	Forsythoside B
S5625	S8261	S5658	S8321	S5698	S8538	S5754	S8660	S5845	S8772
Ammonium lactate	GSK583	Omeprazole Sodium	MLR-1023	Ketorolac tromethamine salt	PF-06651600	Baricitinib phosphate	GI254023X	Chalcone	LY 3200882
S7411	S9112	S7516	S9127	S7589	S9148	S7650	S9171	S7741	S9187
Ascomycin (FK520)	Kaempferitrin	Y-320	Carnosol	N6022	Peiminine	Peficitinib (ASP015K, JNJ-54781532)	Harpagoside	SB239063	Phillygenin
S5640	S8270	S5659	S8334	S5700	S8559	S5768	S8663	S5851	S8777
Ethyl caffeate	SRT2183	Emedastine	XMU-MP-1	Phthalylsulfathiazole	INF39	Fenoterol hydrobromide	Takinib	4'-Methoxychalcone	Evobrutinib
S7414	S9113	S7520	S9128	S7605	S9154	S7651	S9172	S7763	S9188
Caffeic Acid Phenethyl Ester	Ononin	Darapladib (SB-480848)	Isoacteoside	Filgotinib (GLPG0634)	Chelidonine	SB225002	Phellodendrine	D-Luciferin	Magnoflorine chloride

S5647	S8291	S5661	S8337	S5711	S8560	S5769	S8677	S5871	S8790
Emedastine Difumarate	Atractylenolide I	Tiaprofenic acid	1400W 2HCl	Deracoxib	Seletalisib (UCB-5857)	Fenoterol	Cu-CPT22	Trans-Tranilast	ML385
S7429	S9115	S7523	S9129	S7608	S9157	S7656	S9173	S7775	S9199
MI-2 (MALT1 inhibitor)	Wogonoside	Entospletinib (GS-9973)	20(S)-Ginsenoside Rh1	UM171	Neoandrographolide	CPI-360	Sec-O-Glucosylhamaudol	Emricasan	Pseudoginsenoside F11
S5904	S8813	S5950	S9032	S7046	S9047	S7167	S9064	S7215	S9085
WHI-P97	LIT-927	Fingolimod	Sanguinarine	Brefeldin A	Leonurine Hydrochloride	SSR128129E	Trilobatin	Losmapimod (GW856553X)	Corynoline
S7792	S9200	S7864	S9224	S7936	S9240	S8034	S9254	S8126	S9288
SRT2104 (GSK2245840)	Pneumocandin B0	Oltipraz	Dehydroandrographolide Succinate	KD025 (SLx-2119)	Isofraxidin	Apremilast (CC-10004)	Skimmin	MK571	Casticin
S5905	S9013	S6005	S9033	S7051	S9050	S7171	S9065	S7218	S9088
Suberohydroxamic acid	Paulownin	VX-702	Incensole acetate	CGI1746	Boldine	GKT137831	Songorine	Alvelestat (AZD9668)	Calycosin-7-O-beta-D-glucoside
S7799	S9203	S7867	S9225	S7937	S9241	S8044	S9260	S8133	S9289
Pexmetinib (ARRY-614)	Notopterol	Oleuropein	Santalol	Nemiralisib (GSK2269557)	Polygalaxanthone III	BMS-345541	Nodakenin	Resiquimod	Astragalin
S5909	S9017	S7006	S9035	S7080	S9051	S7173	S9068	S7262	S9089
Anagliptin	Columbianadin	BAY-61-3606	Isovitexin	RN486	Asperulosid	Spebrutinib (CC-292, AVL-292)	Trillin	Vidofludimus	Magnoflorine
S7812	S9213	S7892	S9227	S7938	S9242	S8049	S9264	S8136	S9290
Itacitinib (INCB39110)	Jujuboside A	Avadomide(CC-122)	Sinensetin	GSK2292767	Narirutin	Tubastatin A	Decursin	Sivelestat (ONO-5046)	Linarin
S5917	S9019	S7007	S9037	S7113	S9052	S7174	S9071	S7263	S9090
Solcitinib	Quillaic acid	Binimetinib (MEK162, ARRY-162, ARRY-438162)	Tenacissoside H	Zebularine	Auraptene	Opaganib (ABC294640)	Nootkatone	AZD1981	Corilagin
S7813	S9216	S7900	S9228	S7952	S9243	S8067	S9269	S8141	S9293
AMG319	Esculentoside A	SW033291	Schisandrin C	Ozanimod (RPC1063)	Protosappanin B	Vorapaxar	Epmedin C	Cl-amidine	Isoliquiritin
S5925	S9022	S7015	S9038	S7125	S9053	S7179	S9072	S7273	S9091
Olodaterol hydrochloride	20S-Ginsenoside Rg3	Birinapant	Calycosin	KPT-185	Irisflorentin	BAF312 (Siponimod)	Sweroside	SC75741	10-Gingerol
S7818	S9217	S7911	S9229	S7953	S9245	S8078	S9272	S8165	S9296
Pexidartinib (PLX3397)	Complanatuside	PD-1/PD-L1 inhibitor 1	Linderane	ETC-1002	Phellodendrine chloride	Bardoxolone Methyl	Koumine	Cucurbitacin B	Fraxin
S5928	S9024	S7018	S9039	S7156	S9054	S7188	S9074	S7286	S9092
Zerumbone	AKBA	CZC24832	Albiflorin	Marimastat (BB-2516)	Pectolinarin	CID755673	Buddlejasaponin IVb	RO9021	Norisoboldine
S7835	S9220	S7912	S9235	S8009	S9247	S8097	S9280	S8166	S9297
I-BRD9	Stylopine hydrochloride	BMS202 (PD-1/PD-L1 inhibitor 2)	Didymin	AG-18	Isoliensinine	C-DIM12	Demethoxycurcumin	ONO-4059 (GS-4059) hydrochloride	Kirenol
S5929	S9028	S7028	S9043	S7161	S9057	S7189	S9083	S7300	S9095
4-Octyl Itaconate	Cimifugin	Duvelisib (IPI-145, INK1197)	6-Shogaol	Motolimod (VTX-2337)	Sinigrin	I-BET-762	Rhapontin	PJ34 HCl	Senegenin
S7851	S9221	S7929	S9236	S8016	S9248	S8124	S9281	S8178	S9298
AZD3264	Ferulaldehyde	BEC HCl	α-Cyperone	Vonoprazan Fumarate (TAK-438)	Homoorientin	BMS-582949	Peimine	Wnt agonist 1	Corylin
S5935	S9029	S7038	S9044	S7162	S9061	S7211	S9084	S7304	S9098
Alvimopan	Prim-o-glucosylcimifugin	Epoxomicin	Ginsenoside F2	Mdivi-1	Hydroxy safflor yellow A	PF-04418948	Rhoifolin	CPI-203	Dehydrodiisoeugenol
S7856	S9222	S7935	S9237	S8032	S9252	S8125	S9287	S8182	S9300
Tenofovir Alafenamide (GS-7340)	Dipotassium glycyrrhizinate	Y-39983 HCl	Berberrubine	PRT062607 (P505-15, BIIB057) HCl	Corydaline	Pamapimod (R-1503, Ro4402257)	Genkwanin	NSC87877	Methylprotodioscin
S9304	S9354	S9407	S9430	S9472	S9507	S6136	S1205	S2620	S9067
Platycodin D	Oxalic acid	Angoroside C	Tomatidine	Oxtriphylline	(−)-Norepinephrine	(±)-α-Bisabolol	PIK-75 HCl	GSK256066	Pseudoprotodioscin
S9309	S9359	S9408	S9431	S9474	S9511	S6185	S1211	S3614	S9246
Micheliolide	Anthraquinone-2-carboxylic Acid	Beta-Elemonic	Dehydrocorydalin	Acetylharpagide	Crocin I	Arachidonic acid	Imiquimod	Lupeol	Tiliroside
S9311	S9366	S9411	S9433	S9481	S9552	S6243	S1353	S3721	S9257
Germacrone	5'-Adenylic acid	Liensinine	Tubeimoside II	Blinin	Farrerol	Ethyl pyruvate	Ketoconazole	Bilastine	Acetylshikonin
S9313	S9371	S9416	S9434	S9484	S9560	S6249	S1986	S3770	S5908
Atractylenolide III	1-Iodoadamantane	Triptonide	8-Gingerol	Darutoside	Anisodamine Hydrobromide	olinic acid (PCL (	Meclizine 2HCl	Sodium Aescinate	Garenoxacin
S9317	S9386	S9417	S9437	S9487	S5504	S6257	S2047	S4218	S6118
Forsythoside A	Polygalacic acid	Homoplantaginin	Echinatin	Praeruptorin C	Ropivacaine	Acetylglucosam	Lornoxicam	Amoxapine	Terpinen-4-ol
S9318	S9403	S9419	S9439	S9488	S6058	S6275	S2206	S4262	S7888
(20R)-Ginsenoside Rh1	Ecliptasaponin A	Paeruptorin B	Kaurenoic acid	Praeruptorin D	β-Caryophyllene	(−)-β-Pinene	R788 (Fostamatinib) Disodium	Ebastine	Spautin-1
S9324	S9405	S9425	S9440	S9502	S6068	S8760	S2292	S7221	S7896
Alnustone	Sesamoside	Trifolirhizin	Naringenin chalcone	Madecassic acid	cycline monohy	erdomide(CC22	Diosmin	GS-9620	Sodium Tauroursodeoxycholate (TUDC)

S9350	S9406	S9428	S9449	S4962	S6073	S8765	S2395	S7756	S8595
Ethyl 4-Methoxycinnamate	Sauchinone	Brazilin	Urocanic acid	Agmatine sulfate	Diclofenac acid	PF-04965842	Rheochrysidin	Indoximod (NLG-8189)	Tat-beclin 1 (Tat-BECN1)
S1236	S1893	S3174	S4157	S5097	S5674	S8415	S9121	S1911	S9010
Leucovorin Calcium Pentahydrate	D-glutamine	L-Arginine HCl (L-Arg)	Chloroquine Phosphate	Methotrexate disodium	Lodoxamide Tromethamine	PACAP 1-38	Irigenin	Disodium Cromoglycate	Bevirimat
S1384	S1939	S3631	S4360	S5252	S5801	S8416	S9133	S5695	S9034
Mizoribine	Levamisole hydrochloride	(S)-Methylisothiourea sulfate	Tolmetin Sodium	Ozagrel sodium	Ala-Gln	PACAP 6-38	Chicoric acid	Icatibant Acetate	Isochlorogenic acid C
S1494	S2416	S3852	S4430	S5412	S5866	S8501	S9244	S8030	S9042
Ralimetinib (LY2228820)	Chondroitin sulfate	L-Theanine	Hydroxychloroquine Sulfate	Loxoprofen Sodium	Gastrodenol	DAPTA	8-O-acetyl shanzhiside methyl ester	Plerixafor (AMD3100)	Wedelolactone
S1516	S2420	S4008	S4606	S5549	S5867	S8522	S9312	S5450	S9081
Cidofovir	Aloperine	Pemirolast potassium	Glutathione	DL-Arginine	Bendazac L-lysine	Compstatin	Cyanidin-3-O-glucoside chloride	Sofalcone	Anemoside B4
S1518	S2507	S4013	S4700	S5591	S7507	S8549	S9315	S5456	S1848
Ibuprofen Lysine	Salbutamol Sulfate	Sodium Monofluorophosphate	4-Aminobutyric acid	Ectoine	LDN-193189 2HCl	AUNP-12	Praeruptorin A	Isobavachalcone	Curcumin
S1648	S2877	S4028	S4704	S5635	S7809	S6322	S4996	S5499	S1876
Cytarabine	L-NAME HCl	Dexamethasone Sodium Phosphate	D-(+)-Cellobiose	L-cysteine	MCC950(CP-456773)	Pipecolic acid	Tavaborole (AN-2690)	Amantadine	alaciclovir HC
S1853	S3013	S4117	S4791	S3910	S8158	S4581	S5009	S5550	S2261
Penicillamine	Plerixafor 8HCl (AMD3100 8HCl)	Histamine Phosphate	S-allyl-L-cysteine	4',7-Dimethoxyisoflavone	PD-1/PD-L1 Inhibitor 3	Triacetin	Brivudine	Ethyl gallate	Andrographolide
S1857	S3146	S4137	S4811	S3971	S8414	S4592	S5036	S5684	S2271
Etidronate	Tripelennamine HCl	Eprazinone 2HCl	VitaMin U	Fusidine	PACAP 1-27	Captisol (SBE-β-CD)	Nisin	Lapachol	Berberine chloride
S1047	S1878	S2313	S2612	S4029	S4198	S4726	S5086	S5757	S2557
stat (SAHA, M	Ganciclovir	Indole-3-carbinol	Ribitol	Colistin Sulfate	Aminothiazole	Lauric Acid	p-Anisaldehyde	Isoeugenol	Terbinafine HCl
S1282	S1883	S2321	S3007	S4050	S4254	S4759	S5099	S5924	S2597
Artemisinin	Idoxuridine	Magnolol	Zanamivir	Valganciclovir HCl	Apramycin Sulfate	p-Coumaric Acid	4-Aminophenylarsonic acid	Olanexidine Hydrochloride semihydrate	Oseltamivir Phosphate
S1573	S1962	S2334	S3073	S4073	S4518	S4843	S5312	S6067	S3801
udil (HA-1077)	Sulphadimethoxine	Oleanolic Acid	Caspofungin Acetate	Sodium 4-Aminosalicylate	Chloroxylenol	Potassium acetate	Urolithin A	2,4-dichlorobenzyl alcohol	Sodium Houttuyfonate
S1644	S1964	S2467	S3622	S4172	S4547	S4885	S5390	S4564	S3860
Nitrofural	Rimantadine	Famciclovir	Diammonium Glycyrrhizinate	Cetylpyridinium Chloride	8-Hydroxyquinoline	Taurolidine	N-(Hytroxymethy)micotinamide	Diethylcarbamazine citrate	Allicin
S1807	S2120	S2486	S3695	S4184	S4550	S4942	S5440	S4573	S4959
Aciclovir	Arbidol HCl	Moroxydine HCl	Cystamine dihydrochloride	Penciclovir	Azelaic acid	4-Hydroxy-3,5-dimethoxybenzyl alcohol	Berberine Sulfate	m-Tolyl acetate	Skatole
S1833	S2259	S2504	S3746	S4196	S4559				
oconazole nit	Aloe-emodin	Ribavirin	Lumefantrine	Ethacridine lactate monohydrate	Cloxiquine				
