# Supplementary figures and images for: High-Throughput NanoBiT-Based Screening for Inhibitors of HIV-1 Vpu and Host BST-2 Protein Interaction
Source: Int J Mol Sci. 2021 Aug 27;22(17):9308. doi: 10.3390/ijms22179308 (PMC8431494; doi:10.3390/ijms22179308)

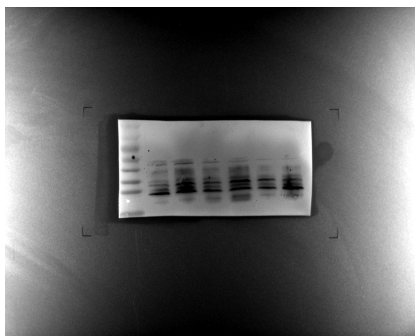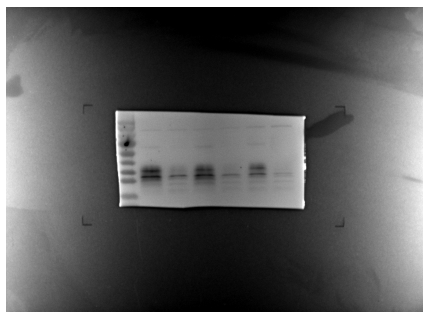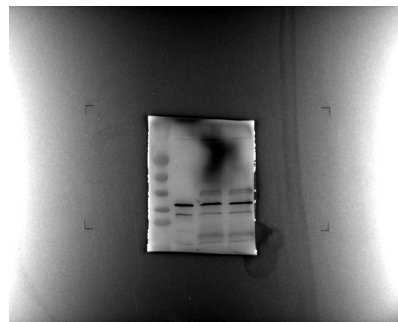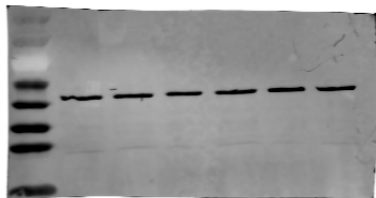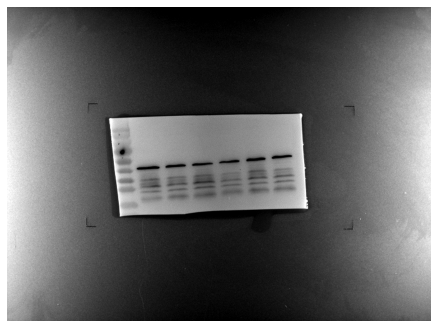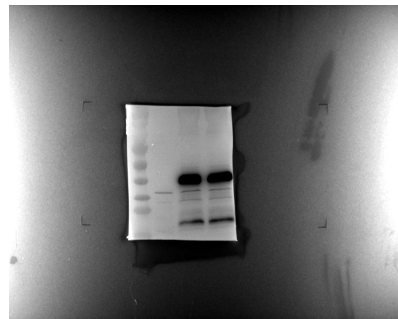

Supplement: Supplementary file 1 [file ijms-22-09308-s001.zip › ijms-1323972-supple-revised/blots.pdf]
